# FSH protects mouse granulosa cells from oxidative damage by repressing mitophagy

**DOI:** 10.1038/srep38090

**Published:** 2016-11-30

**Authors:** Ming Shen, Yi Jiang, Zhiqiang Guan, Yan Cao, Shao-chen Sun, Honglin Liu

**Affiliations:** 1 College of Animal Science and Technology, Nanjing Agricultural University, Nanjing 210095, China

## Abstract

Oxidative stress has been implicated in triggering granulosa cell (GC) death during follicular atresia. Recent studies suggested that follicle-stimulating hormone (FSH) has a pivotal role in protecting GCs from oxidative injury, although the exact mechanism remains largely unknown. Here, we report that FSH promotes GC survival by inhibiting oxidative stress-induced mitophagy. The loss of GC viability caused by oxidative stress was significantly reduced after FSH treatment, which was correlated with impaired activation of mitophagy upon oxidative stress. Compared with FSH treatment, blocking mitophagy displayed approximate preventive effect on oxidative stress-induced GC death, but FSH did not further restore viability of cells pretreated with mitophagy inhibitor. Importantly, FSH suppressed the induction of serine/threonine kinase PINK1 during oxidative stress. This inhibited the mitochondrial translocation of the E3 ligase Parkin, which is required for the subsequent clearance of mitochondria, and ultimately cell death via mitophagy. In addition, knocking down PINK1 using RNAi confirmed the role of the FSH-PINK1-Parkin-mitophagy pathway in regulating GC survival under oxidative conditions. These findings introduce a novel physiological function of FSH in protecting GCs against oxidative damage by targeting PINK1-Parkin-mediated mitophagy.

In mammalian ovaries, follicular atresia is a common occurrence that destroys more than 99% of the ovarian follicles^1^,^2^. Previous studies revealed a close correlation between oxidative stress-induced granulosa cell (GC) injury and the onset of follicular atresia^3^. For example, radiation, smoking, alcohol, and pathologic conditions including obesity and malnutrition produce high reactive oxygen species (ROS) levels in ovarian follicles, leading to oxidative stress and extensive GC damage, followed by intensified follicular atresia and subsequent ovulation disorders, such as polycystic ovary syndrome and premature ovarian failure^4^. Therefore, elucidating the mechanisms of oxidative stress-induced GC death may provide plausible treatment strategies for reproductive failure related to inappropriate follicular atresia.

Ovarian function is precisely regulated by a complex set of gonadotropins and local ovarian factors. Follicle stimulating hormone (FSH) is a glycoprotein polypeptide hormone secreted by anterior pituitary gland. It stimulates GCs to produce estrogen^5^, promotes the development of antral follicles^6^,^7^,^8^, and determines the selection and dominance of preovulatory follicles^9^. Accumulating evidence suggests that FSH plays a critical role in maintaining the well-being of antral follicles by repressing GC death^10^,^11^,^12^. However, few reports have evaluated the prosurvival effects of FSH on GCs undergoing oxidative stress. Although our previous data indicate a potential function for FSH in antagonizing stress-induced GC injury^13^, its targets and the underlying mechanism by which it protects GCs are still not fully understood.

GC apoptosis was traditionally considered the hallmark of follicular atresia^14^. However, recent studies have provided evidence that other forms of programmed cell death (PCD) such as autophagy can be activated, mainly in GCs, during follicular atresia^15^,^16^. These observations were further supported by reports that GC death could be induced by oxidized LDL (oxLDL)-dependent lectin-type oxLDL receptor (LOX1)-activated autophagy^17^,^18^. Specifically, obese women with high levels of oxLDL displayed increased oxidative stress in the ovaries, which led to a higher rate of anovulatory infertility^17^. This raises the question of whether the ability of FSH to protect against oxidative damage is related to the suppression of autophagic GC death.

Several forms of autophagy that lead to the degradation of specific organelles have been described, including ribophagy for ribosome-targeted degradation^19^, mitophagy for mitochondria-targeted degradation^20^, pexophagy for the selective degradation of peroxisomes^21^, and ER-phagy or reticulophagy for endoplasmic reticulum-specific degradation^22^. Emerging evidence suggests that oxidative stress-induced mitochondrial permeability transition is responsible for mitochondrial membrane potential (Δψm) depolarization, which results in the accumulation of PTEN-induced kinase 1 (PINK1) on the outer mitochondrial membrane. PINK1 then recruits the E3 ubiquitin ligase Parkin to initiate the autophagic degradation of damaged mitochondria^23^,^24^,^25^. However, mitophagic signaling pathways have distinct functions depending on the cell types or environmental stimuli. Few reports have systematically investigated the potential involvement and molecular mechanisms of mitophagy in the FSH-mediated protection of GCs from oxidative damage. The current study examined the effects of FSH treatment on cell viability, mitochondrial integrity, mitophagic flux, and molecular component of mitophagic signaling. The results demonstrated that the suppression of mitophagy plays a critical role in FSH-mediated oxidative damage protection in GCs through a PINK1-Parkin-dependent manner.

## Results

### FSH inhibits the activation of granulosa cell (GC) autophagy during oxidative stress

**Figure 1:**
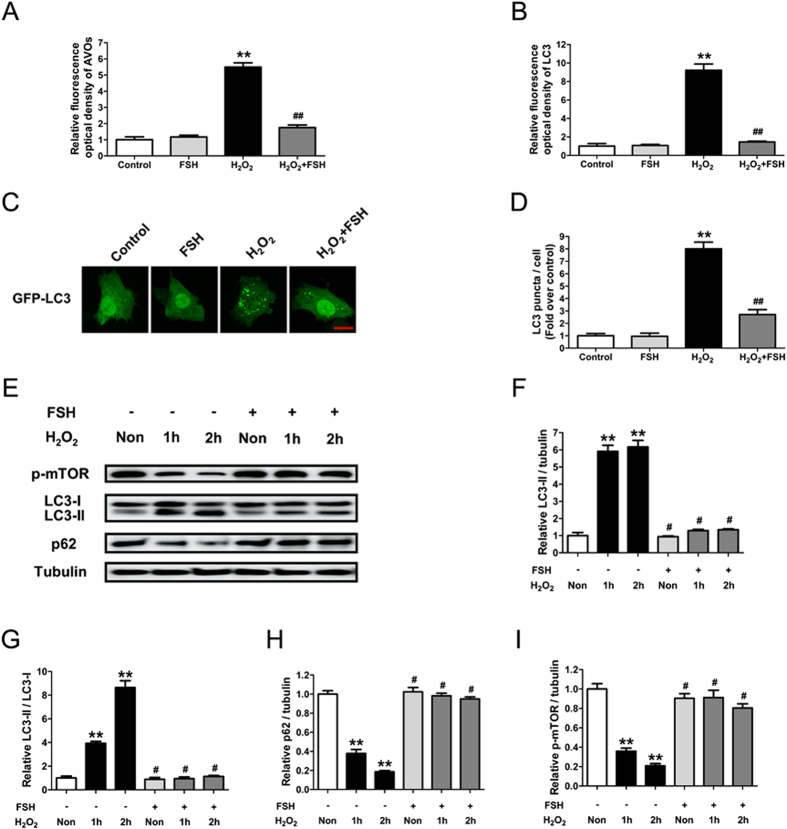
FSH reduced autophagy in GCs upon oxidative stress. Primary cultured GCs with 1 h of H_2_O_2_ incubation (200 μM) were treated with 7.5 IU/ml FSH for 2 h. The acidic autophagic vacuoles were detected using acridine orange staining, and the LC3 protein was counterstained with anti-LC3. The formation of autophagic vacuoles was quantified by calculating the optical density of AVOs (**A**) and LC3 (**B**) per cell. Data represent mean ± S.E; n = 3 in each group. **Represents P < 0.01 compared to control group. ^##^Represents P < 0.01 compared to H_2_O_2_ group. (**C**) GCs transfected with GFP-LC3 plasmid for 48 h were incubated with 200 μM H_2_O_2_ for 1 h and cultured for another 2 h in the presence of 7.5 IU/ml FSH. Laser confocal-scanning microscopy was employed to observe the GFP fluorescent puncta in GCs. Bar, 10 μm. (**D**) Quantification of the GFP-LC3 puncta per cell. Experiments were repeated in triplicate, and three fields of each coverslip were selected in random for counting. Data represent mean ± S.E; n = 3. **P < 0.01 vs. control group; ^##^P < 0.01 vs. H_2_O_2_ group. (**E**) GCs incubated with or without 200 μM H_2_O_2_ for 1 h were then treated with FSH for 1–2 h. The expression of LC3, p62, and p-mTOR in GCs was determined by western blotting. (**F–I**) Quantification of LC3-II expression, conversion of LC3-I to LC3-II, p62 degradation and mTOR dephosphorylation. α-tubulin served as the control for loading. Data represent mean ± S.E; n = 3. **Represents P < 0.01 compared to control group. ^#^Represents P > 0.05 compared to control group.

To determine whether FSH affects GC autophagy during oxidative stress, acridine orange staining was employed to visualize the production of acidic autolysosomes. As shown in Supplementary Fig. 1 and Fig. 1A, H_2_O_2_ treatment markedly elevated the number of autolysosomes in cultured GCs, which was significantly blocked by FSH. To confirm this finding, the effects of FSH on LC3, a hallmark of autophagy, were analyzed. Immunofluorescence staining using anti-LC3 antibodies revealed that treating GCs with FSH strongly attenuated the oxidative stress-induced expression of LC3 (Supplementary Fig. 1 and Fig. 1A), which was colocalized with the acidic autophagic vacuoles (Supplementary Fig. 1). Thus, these data suggest that FSH suppresses autophagosome formation. The ability of FSH to inhibit autophagy was also demonstrated by monitoring GCs that had been transfected with a vector expressing GFP-LC3. Cells pre-treated with H_2_O_2_ displayed a dramatic decrease in the number of GFP-LC3 puncta following FSH incubation compared with the control group (Fig. 1C and D), consistent with the observations from the combined acridine orange and anti-LC3 staining under the same conditions (Supplementary Fig. 1, Fig. 1A and B). Western blotting analysis of autophagy biomarkers, including the accumulation of LC3-II, the conversion of LC3-I to LC3-II, p62 degradation, and mTOR dephosphorylation (Fig. 1E–I), further confirm the inhibitory effects of FSH on oxidative stress-induced autophagy.

### The inhibition of autophagy by FSH protects GCs from oxidative injury

**Figure 2:**
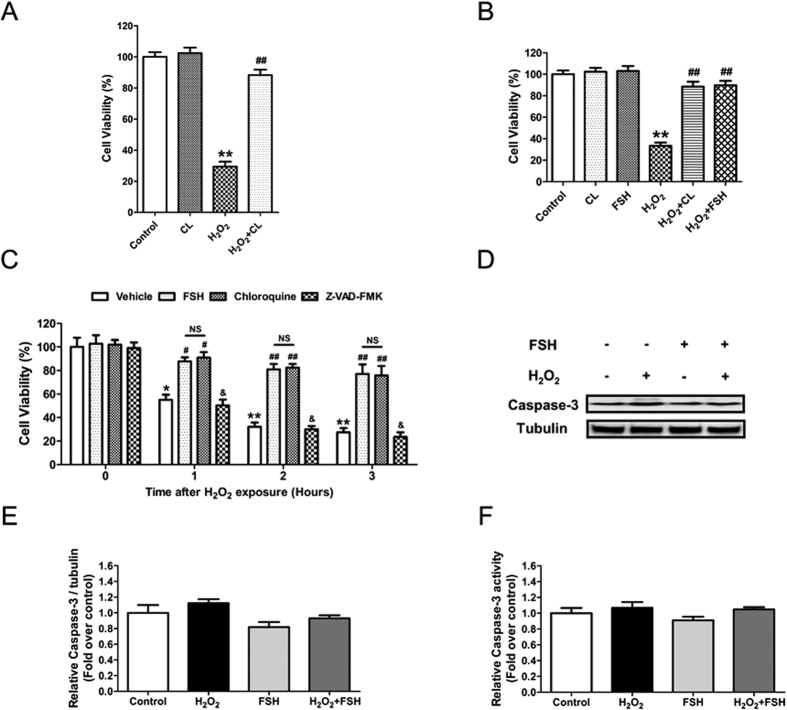
FSH prevented GCs from oxidative injury by inhibiting autophagy. (**A**) Primary cultured GCs with 1 h of H_2_O_2_ incubation (200 μM) were treated with 50 μM chloroquine for 2 h, and cell viability was determined using the CCK-8 assay. Data represent mean ± S.E; n = 3. P** < 0.01 compared with control group. p^##^ < 0.01 compared with H_2_O_2_-only-treated cells. CL, Chloroquine. (**B**) GCs were exposed to 200 μM H_2_O_2_ for 1 h, and then treated with 50 μM chloroquine or 7.5 IU/ml FSH for 2 h. Cell viability was measured by CCK-8 assay. Data represent mean ± S.E; n = 3 in each group. P** < 0.01 compared with control group. p^##^ < 0.01 compared with H_2_O_2_-only-treated cells. (**C**) GCs incubated with 200 μM H_2_O_2_ for 1 h were treated with 7.5 IU/ml FSH, 50 μM chloroquine, or 50 μM Z-VAD-FMK. 0, 1, 2 or 3 h later, cell viability was detected using the CCK-8 assay. Data represent mean ± S.E; n = 3. *Represents P < 0.05 compared to vehicle group at 0 h. **Represents P < 0.01 compared to vehicle group at 0 h. ^#^Represents P < 0.05 compared to H_2_O_2_-only-treated cells. ^##^Represents P < 0.01 compared to H_2_O_2_-only-treated cells. &Represents P > 0.05 compared to H_2_O_2_-only-treated cells. NS, not significant, P > 0.05. (**D**) GCs incubated with 200 μM H_2_O_2_ for 1 h were then treated with or without FSH for 2 h. The expression of caspase-3 in GCs was determined by western blotting. α-tubulin served as the control for loading. (**E**) Quantification of relative caspase-3 protein levels using densitometric analysis. Data represent mean ± S.E; n = 3. (**F**) The detection of caspase-3 activity in GCs with the indicated treatments. Data represent mean ± S.E; n = 3.

To investigate the effects of autophagy on GC death upon oxidative stress, the cell viability of H_2_O_2_-treated GCs was examined in the presence of the autophagy inhibitor chloroquine. As shown in Fig. 2A, the loss of GC viability caused by H_2_O_2_ exposure was significantly reduced by chloroquine treatment. Consistent with this, FSH revealed similar level of suppression in H_2_O_2_-induced cell death (Fig. 2B). The results indicate the involvement of autophagy in FSH-mediated cellular resistance to oxidative injury. Since our previous results showed that chronic H_2_O_2_ exposure triggered apoptosis in GCs^26^, we next tested whether FSH-mediated autophagy correlated with apoptosis in the early stages of H_2_O_2_-induced cell death. Both FSH and chloroquine significantly repressed the downregulation of GC viability by oxidative stress; however, the caspase inhibitor Z-VAD-FMK did not affect the viability of H_2_O_2_-treated cells (Fig. 2C). Consistent with this, western blotting and caspase-3 activity analysis showed no significant change in caspase-3 induction following FSH treatment (Fig. 2D and E). These findings suggested that the FSH-induced suppression of autophagic cell death in H_2_O_2_-treated GCs might be independent of apoptosis.

### FSH protects mitochondria from oxidative damage in GCs

**Figure 3:**
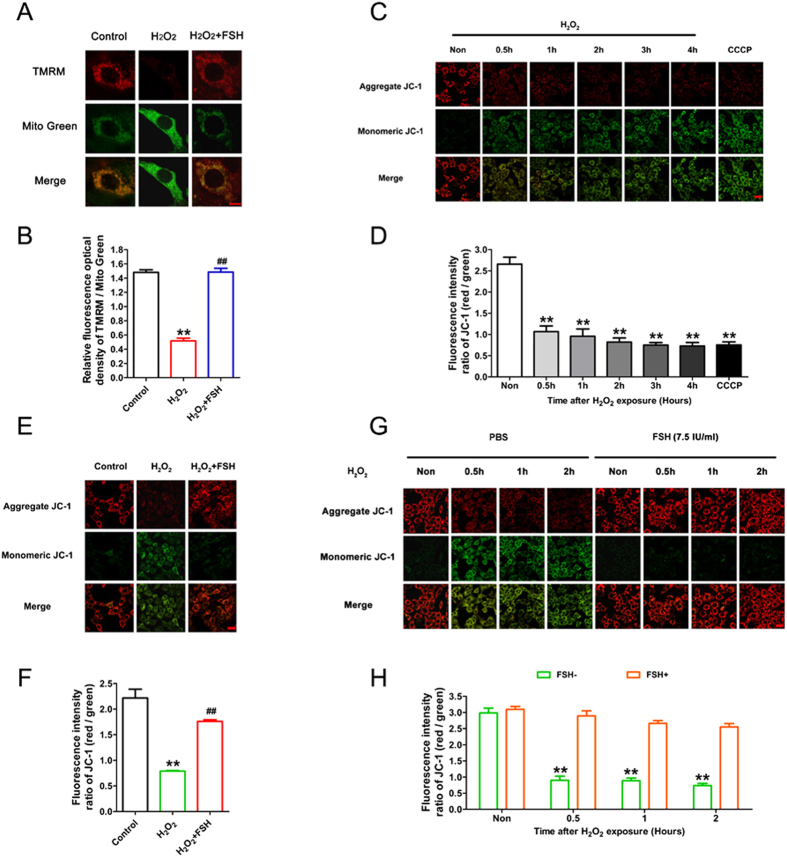
FSH maintained mitochondrial integrity in GCs subjected to oxidative stress. (**A**) GCs harvested from dominant follicles were exposed to 200 μM H_2_O_2_ for 1 h, and then treated with 7.5 IU/ml FSH for 2 h. Mitochondria were double-stained with TMRM (red) and Mito Green (green), and observed using laser confocal-scanning microscopy. Bar, 10 μm. (**B**) Mitochondrial membrane potential (Δψm) was evaluated by calculating the relative fluorescence intensity of TMRM/Mito green. Data represent mean ± S.E; n = 3. Significances were marked as **P < 0.01 vs. control group; ^##^P < 0.01 vs. H_2_O_2_ group. (**C**) GCs incubated with 200 μM H_2_O_2_ for 1 h were cultured in DMEM/F-12 medium for another 0–4 h before collection. Cells were then stained with JC-1 to visualize the healthy mitochondria (uptaking red fluorescent aggregates of JC-1) and defective mitochondria (uptaking green fluorescent monomeric JC-1). The treatment of cells with 50 mM CCCP for 20 min was used as a positive control. Bar, 20 μm. (**D**) The fraction of depolarized mitochondria was evaluated by measuring the fluorescence intensity of aggregate JC-1/monomeric JC-1. Data represent mean ± S.E; n = 3. P** < 0.01 compared with control group (0 h group). (**E**) JC-1 staining image of GCs treated with 7.5 IU/ml FSH for 3 h following 1 h of H_2_O_2_ (200 μM) incubation. Bar, 20 μm. (**F**) The quantitative fluorescence intensity of aggregate JC-1/monomeric JC-1 as shown in (**E**). Data represent mean ± S.E; n = 3. P** < 0.01 compared with control group. p^##^ < 0.01 compared with H_2_O_2_-only-treated cells. (**G**) GCs incubated with or without 200 μM H_2_O_2_ for 1 h were then treated with FSH for 0.5–2 h. Cells were collected for JC-1 staining. Bar, 20 μm. (**H**) The quantitative fluorescence optical density of aggregate JC-1/monomeric JC-1 as shown in (**G**). Data represent mean ± S.E; n = 3. P** < 0.01 compared with control group.

Recent studies suggested that mitophagy might function as an effector of programmed cell death^27^,^28^. To further elucidate the mechanisms by which FSH protects against oxidative injury, we next investigated whether FSH is required for mitochondrial integrity in GCs exposed to oxidative stress. Using double staining of tetramethylrhodamine ethyl ester (TMRM) and MitoTracker Green (Mito Green), we first evaluated the effects of FSH on mitochondrial membrane potential (Δψm) in H_2_O_2_-treated GCs by calculating the ratio of TMRM/Mito green fluorescence intensities. As shown in Fig. 3A and B, H_2_O_2_ treatment increased the fraction of depolarized mitochondria, which was significantly blocked by FSH. To better assess mitochondrial dysfunction, GCs were next stained with JC-1, a more specific dye for examining defective mitochondria. Since red fluorescent aggregates of JC-1 (J-aggregates) released from damaged mitochondria tend to form green fluorescent JC-1 monomers in the cytosol^29^, the ratio between green and red JC-1 fluorescence was used to estimate mitochondria integrity in our current study. As shown in Fig. 3C–F, GCs incubated with H_2_O_2_ exhibited a marked dose-dependent decrease in functional mitochondria. In contrast, FSH significantly suppressed mitochondrial injury and restored mitochondrial membrane potential (Δψm) following oxidative stress. Notably, FSH effectively mitigated mitochondrial injury within 1–2 hours of H_2_O_2_ exposure (Fig. 3G and H), consistent with the time courses of oxidative stress-induced autophagic response to FSH treatment in GCs (Fig. 1). Therefore, these data suggest that FSH treatment of GCs might protect mitochondria from oxidative damage in a mitophagy-dependent manner.

### FSH inhibits oxidative stress-induced mitophagy in GCs

**Figure 4:**
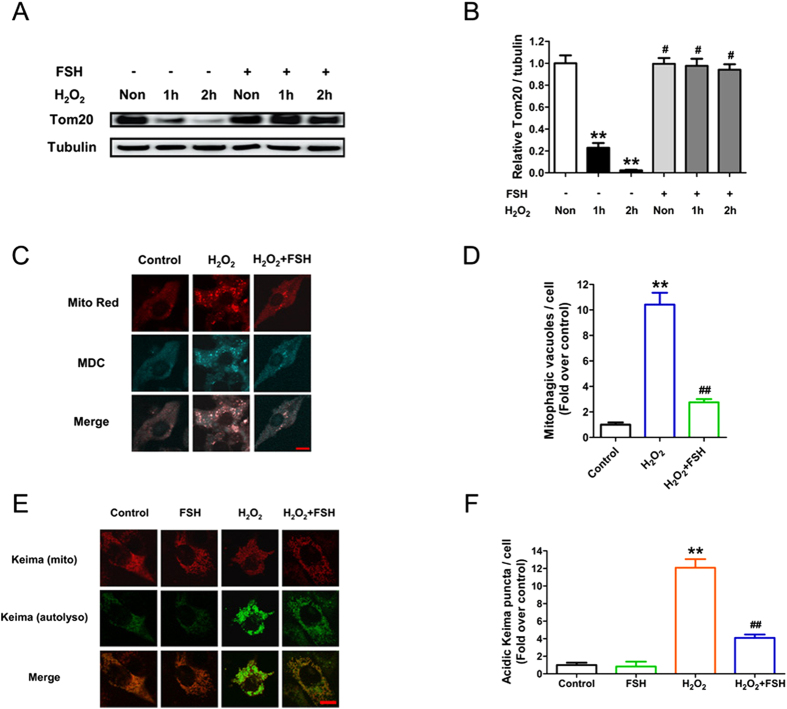
FSH suppressed mitophagy in GCs suffering from oxidative stress. (**A**) GCs incubated with or without 200 μM H_2_O_2_ for 1 h were then treated with FSH for 1–2 h. The protein level of Tom20 was determined by western blotting. (**B**) Quantification of immunoblot signal for Tom20. α-tubulin served as the control for loading. Data represent mean ± S.E; n = 3. **Represents P < 0.01 compared to control group. ^#^Represents P > 0.05 compared to control group. (**C**) GCs were exposed to 200 μM H_2_O_2_ for 1 h, and then treated with 7.5 IU/ml FSH for 2 h. Mitochondria were labeled using Mito Red (red), and the autophagic vacuoles were counterstained with MDC (cyan). Bar, 10 μm. (**D**) The number of the mitophagic vacuoles per cell was counted. Data represent mean ± S.E of at least 30 cells from 3 separate experiments. Significances were marked as **P < 0.01 vs. control group; ^##^P < 0.01 vs. H_2_O_2_ group. (**E**) GCs transfected with pMT-mKeima-Red for 24 h were incubated with 200 μM H_2_O_2_ for 1 h and cultured for another 3 h in the presence of 7.5 IU/ml FSH. Laser confocal-scanning microscopy was employed to observe the mitochondria-localized Keima (red; pseudo coloured) and the acidic Keima puncta (green; pseudo coloured) delivered within autolysosomes. Bar, 10 μm. (**F**) Quantification of the acidic Keima per cell. Experiments were repeated in triplicate, and three fields of each coverslip were selected in random for counting. Data represent mean ± S.E; n = 3. Significances were marked as **P < 0.01 vs. control group; ^##^P < 0.01 vs. H_2_O_2_ group.

To verify whether mitophagy is correlated with FSH-mediated GC survival following oxidative stress, we next evaluated the amount of mitochondria and their distribution in autophagic vacuoles. Using immunoblotting of the mitochondrial marker protein Tom20 for quantifying the mitochondrial mass, we observed that FSH significantly restored Tom20 levels in GCs pre-treated with H_2_O_2_, suggesting a role of FSH in suppressing oxidative stress-induced mitochondrial clearance (Fig. 4A and B). Next, GCs were co-stained with MDC and Mito Red to visualize the localization of mitochondria in autophagic vacuoles and assess if mitophagy affects the elimination of mitochondria. As shown in Fig. 4C and D, FSH treatment markedly attenuated the aggregation of mitochondria in MDC-labeled vesicles upon H_2_O_2_ stimulation, suggesting that FSH might inhibit the formation of mitophagic vacuoles. To validate the fusion between these mitochondria-containing vesicles and lysosomes, GCs were transfected with an expression plasmid containing mitochondria-targeted Keima. Keima is a pH-sensitive protein that fluoresces at 440 nm (neutral pH) in the cytoplasm or mitochondrial matrix and 586 nm in the acidic conditions found in autolysosomes^30^. As shown in Fig. 4E and F, FSH remarkably reduced the number of acidic Keima puncta in H_2_O_2_-treated GCs, suggesting that FSH blocked the incorporation of mitophagic vacuoles into lysosomes. Based on these data, we proposed that mitophagy might contribute to FSH-mediated GC survival in response to oxidative injury.

### FSH counteracts oxidative injury in GCs by inhibiting mitophagy

**Figure 5:**
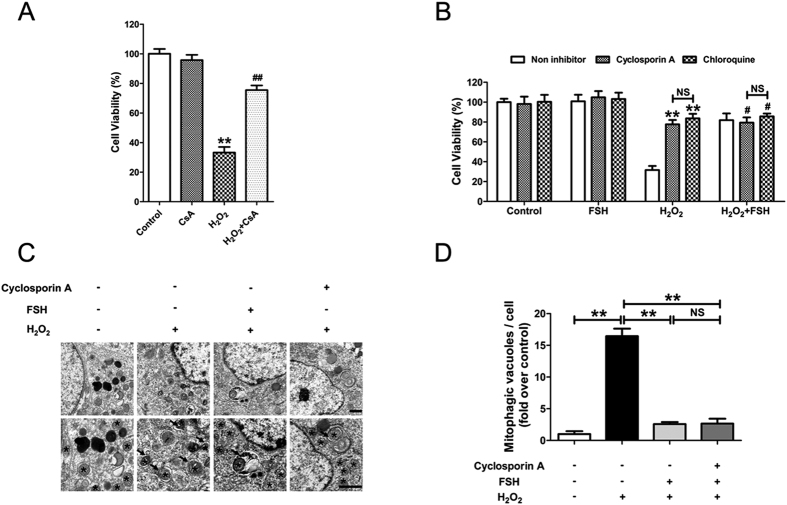
FSH-mediated suppression of mitophagy contributed to GC survival in response to oxidative injury. (**A**) Primary cultured GCs with 1 h of H_2_O_2_ incubation (200 μM) were treated with 10 μM Cyclosporin A for 2 h, and cell viability was determined using the CCK-8 assay. Data represent mean ± S.E; n = 3. P** < 0.01 compared with control group. p^##^ < 0.01 compared with H_2_O_2_-only-treated cells. CsA, Cyclosporin A. (**B**) GCs incubated with or without 200 μM H_2_O_2_ for 1 h were then treated with 10 μM Cyclosporin A or 50 μM chloroquine in the presence or absence of 7.5 IU/ml FSH. 2 h later, cell viability was detected using the CCK-8 assay. Data represent mean ± S.E; n = 3. **Represents P < 0.01 compared to ‘H_2_O_2_ without inhibitor’ condition. ^#^Represents P > 0.05 compared to ‘H_2_O_2_ + FSH without inhibitor’ condition. NS, not significant, P > 0.05. (**C**) GCs pretreated with 200 μM H_2_O_2_ for 1 h were cultured in medium containing 10 μM Cyclosporin A or 7.5 IU/ml FSH. 1 h later, cells were collected for TEM imaging of the mitophagic vacuoles. Bar, 1 μm. Enlarged images (below) show clearer autophagic vacuoles (arrows) and mitochondria (*). (**D**) Number of mitophagic vacuoles per cell section in GCs. Data represent mean ± S.E; n = 3. **P < 0.01; NS, not significant, P > 0.05.

To further assess whether mitophagy affected the pro-survival actions of FSH under oxidative stress conditions, GCs were treated with the mitophagy inhibitor cyclosporine A (CsA)^31^,^32^. As shown in Fig. 5A, the H_2_O_2_-induced loss of viability in GCs was significantly reduced following CsA treatment. In accordance with this, FSH and chloroquine displayed similar preventive effects on GC death upon oxidative stress. However, FSH could not further increase the viability of cells pretreated with the mitophagy inhibitor CsA (Fig. 5B). The results indicate that the suppression of mitophagy might play a role in FSH-mediated cellular resistance to oxidative damage. To investigate this possibility, we took transmission electron microscope (TEM) images of H_2_O_2_-incubated GCs in the presence of FSH or CsA (Fig. 5C). Mitophagic structures were characterized by the engulfment of mitochondria into double membranous autophagic vacuoles^33^. It was observed that both FSH and CsA strongly inhibited the accumulation of mitochondria-containing vesicles in GCs exposed to oxidative stress (Fig. 5C and D). Therefore, the suppression of mitophagic cell death is required for FSH-induced GC survival against oxidative injury.

### FSH protects against oxidative stress-induced mitophagic GC death via the PINK1-Parkin pathway

**Figure 6:**
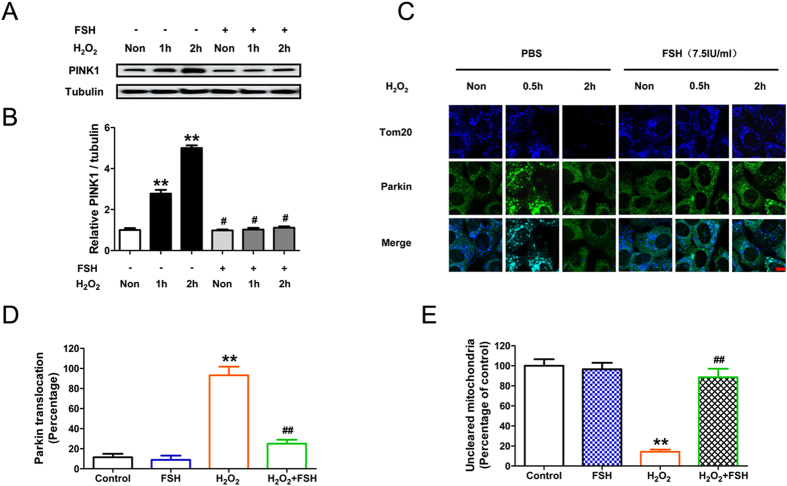
FSH inhibited mitophagy induction by oxidative stress through a PINK1-Parkin-dependent mechanism in GCs. (**A**) GCs incubated with or without 200 μM H_2_O_2_ for 1 h were then cultured in medium containing 7.5 IU/ml FSH for 1–2 h. The expression of PINK1 was determined by western blotting. (**B**) Quantification of relative PINK1 protein levels using densitometric analysis. α-tubulin served as the controlfor loading. Data represent mean ± S.E; n = 3. **Represents P < 0.01 compared to control group. ^#^Represents P > 0.05 compared to control group. (**C**) GCs pretreated with or without 200 μM H_2_O_2_ for 1 h were then grown in medium containing 7.5 IU/ml FSH for another 0.5 or 2 h before collection. Mitochondria were labeled by anti-Tom20 (pseudo coloured; blue) and the Parkin protein was counterstained with anti-Parkin (green). Bar, 20 μm. (**D**) Quantification of Parkin colocalized with defective mitochondria after 0.5 h of H_2_O_2_ treatment. Data represent mean ± S.E; n = 3. (**E**) The amount of uncleared mitochondria after 2 h of H_2_O_2_ treatment is shown as the percentage of Tom20 fluorescence intensity in control group. Data represent mean ± S.E; n = 3. Significances were marked as **P < 0.01 vs. control group; ^##^P < 0.01 vs. H_2_O_2_ group.

**Figure 7:**
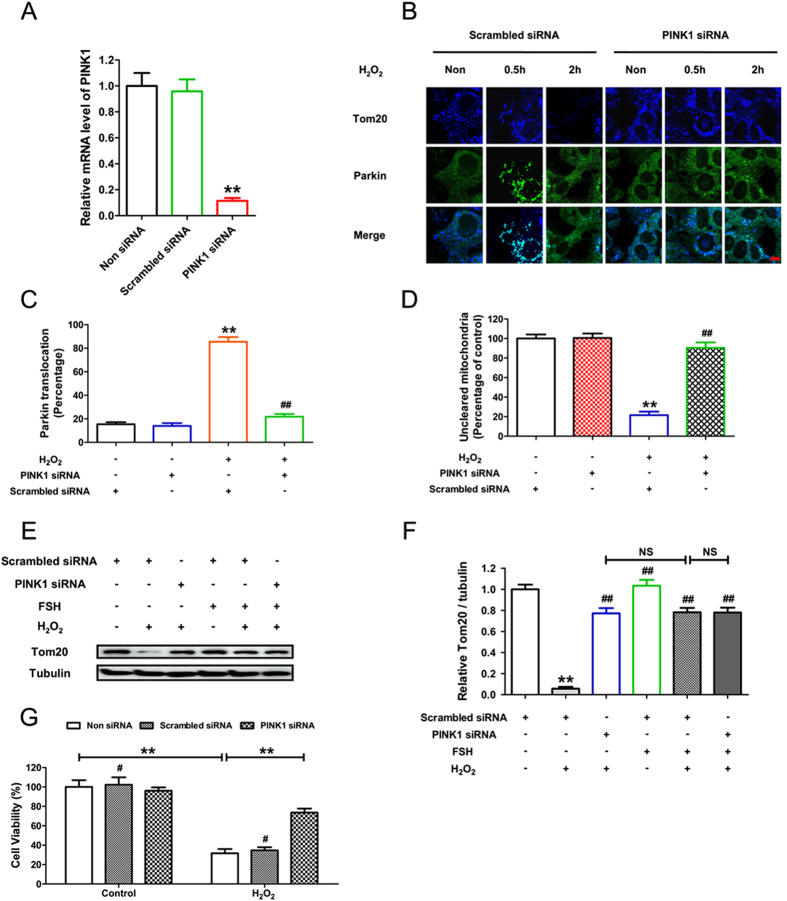
FSH protected GCs from oxidative injury via suppressing the PINK1-Parkin-mitophagy axis. (**A**) Primary cultured GCs remained as an untreated control or were transfected with PINK1 siRNA or scrambledcontrol siRNA for 24 h. The expression of PINK1 mRNA in GCs was determined by qRT-PCR. Expression data were normalized to that of β-actin. (**B**) GCs transfected with PINK1 siRNA or scramble control siRNA for 24 h were incubated with 200 μM H_2_O_2_ for 1 h and then cultured in media containing 7.5 IU/ml FSH for another 0.5 or 2 h before collection. Mitochondria were labeled by anti-Tom20 (pseudo coloured; blue) and the Parkin protein was counterstained with anti-Parkin (green). Bar, 20 μm. (**C**) Quantification of Parkin translocation to injured mitochondria after 0.5 h of H_2_O_2_ treatment. Data represent mean ± S.E; n = 3. (**D**) The amount of uncleared mitochondria after 2 h of H_2_O_2_ treatment is shown as the percentage of Tom20 fluorescence intensity in control group. Data represent mean ± S.E; n = 3. Significances were marked as **P < 0.01 vs. control group; ^##^P < 0.01 vs. H_2_O_2_ group. (**E**) GCs transfected with PINK1 siRNA or scramble control siRNA for 24 h were exposed to 200 μM H_2_O_2_ for 1 h and then grown in the presence of 7.5 IU/ml FSH. 2 h later, cell lysates were collected to determine the protein levels of Tom20 using western blotting. α-tubulin served as the control for loading. (**F**) Quantification of immunoblot signal for Tom20. Data represent mean ± S.E; n = 3. Significances were marked as **P < 0.01 vs. control group; ^##^P < 0.01 vs. H_2_O_2_ group. NS, not significant, P > 0.05. (**G**) GCs transfected with PINK1 siRNA or scrambled control siRNA for 24 h were exposed to 1 h of H_2_O_2_ incubation (200 μM). After washing in PBS, cells were cultured for another 2 h, and cell viability was then determined using the CCK-8 assay. Data represent mean ± S.E; n = 3. **P < 0.01. ^#^Represents P > 0.05 compared to ‘Non siRNA’ condition.

Emerging evidence has implicated a pivotal role for PINK1 and Parkin in mitochondrial quality control by directing the autophagic clearance of defective mitochondria^34^. However, it remains unknown whether PINK1 and Parkin are correlated with the mechanisms by which FSH protects against oxidative injury. To further clarify how mitophagy works during FSH-mediated GC protection against oxidative stress, we thus investigated the potent functions of PINK1 and Parkin under these conditions. Western blotting revealed that GCs incubated with H_2_O_2_ exhibited a significant time-dependent increase in PINK1 levels, whereas FSH abrogated the induction of PINK1 expression following oxidative stress (Fig. 6A and B). However, no marked changes of Parkin expression were detected in FSH-treated GCs following H_2_O_2_ exposure (Supplementary Fig. 2). It has been reported that the recruitment of Parkin to mitochondria promotes mitophagy^35^, we thus examined whether FSH affected Parkin mitochondrial localization. Dual color immunofluorescence staining with antibodies against Parkin and the mitochondrial membrane protein Tom20 (commonly used as a marker for the loss of mitochondria resulting from mitophagy^25^,^36^) showed that Parkin completely translocated to mitochondria clustered in the perinuclear region 0.5 h after H_2_O_2_ exposure (Fig. 6C and D). 1.5 h later, massive mitochondrial loss (a representative hallmark of mitophagy) and the cytosolic redistribution of Parkin were observed (Fig. 6C and E). In contrast, FSH markedly inhibited the recruitment of Parkin to the mitochondria as well as abolished subsequent clearance of mitochondria (Fig. 6C–E). These results suggested the possibility that FSH prevents mitophagic elimination by repressing the mitochondrial translocation of Parkin. To test whether FSH-induced PINK1 suppression exerted any influence on Parkin-mediated mitophagy, we blocked PINK1 expression using RNA interference. As shown in Fig. 7B–D, knocking down PINK1 also prevented Parkin translocation-triggered mitochondrial clearance upon H_2_O_2_ stimulation. In accordance with this, immunoblotting analysis of Tom20 showed that both PINK1 knockdown and FSH treatment significantly repressed the mitochondrial loss following oxidative stress. However, FSH failed to further restore mitochondrial mass in GCs transfected with PINK1 siRNA (Fig. 7E and F), indicating that the inhibition of PINK1 expression by FSH blocked the mitophagic elimination. Therefore, these data suggest that the suppression of PINK1 and the resultant dissociation of Parkin from mitochondria are required for FSH-mediated inhibition of mitophagy. We next examined whether the PINK1-Parkin pathway affected GC survival under oxidative stress. CCK-8 assay showed that PINK1 knockdown remarkably blocked the decline in GC viability caused by H_2_O_2_ exposure (Fig. 7G). Taken together, these data demonstrate that the suppression of mitophagy via the PINK1-Parkin signaling is an important mechanism of FSH-mediated GC protection against oxidative injury.

## Discussion

Excessive ROS derived from endogenous metabolism or environmental stimuli destroys cellular proteins, lipids and DNA in a phenomenon known as oxidative stress. It is well documented that the harmful effects of oxidative stress play a central role in the pathogenesis of female reproductive disorders^4^. For example, stresses such as radiation and smoking generate high levels of ROS in ovarian follicles, causing GC death and subsequent follicular atresia, which is responsible for anovulatory infertility^4^. FSH was identified as a major survival factor for antral follicles because of its ability to inhibit GC death^11^. Our previous data also revealed that FSH blocks the induction of GC death upon oxidative stress^13^; however, the underlying mechanisms were not fully understood. Here, we show for the first time that FSH ameliorates oxidative injury in GCs by suppressing mitophagy.

The current study demonstrates that FSH (1) maintains mitochondrial integrity, (2) restrains oxidative stress-induced mitophagy, (3) prevents mitophagy-dependent GC death, and (4) protects GCs from mitophagic death by inhibiting the PINK1-Parkin pathway under oxidative stress conditions. Collectively, suppressing mitophagy via the FSH-PINK1-Parkin axis is part of an essential adaptive mechanism to preserve GC survival in response to oxidative stress.

It remains controversial whether autophagy (including mitophagy), not only improves cellular resistance to stress stimuli, but also triggers autophagic cell death via the excessive destruction of intracellular components^37^. In the current study, impaired mitophagy was correlated with elevated GC viability in cells treated with FSH under oxidative conditions. Moreover, compared with FSH treatment, the mitophagy inhibitor CsA exhibited similar level of suppression in oxidative stress-induced GC death; however, FSH did not further restore GC viability when mitophagy was inhibited. Thus, these data suggest that FSH inhibits a lethal form of mitophagy in GCs in response to oxidative damage.

Current knowledge of FSH-induced GC survival is mostly related to the regulation of apoptosis^11^, since apoptotic PCD is traditionally considered the major cause of GC death and follicular atresia^38^,^39^. Indeed, accumulating evidence suggests that autophagy is an alternative form of PCD that mainly occurs in GC layers throughout the antral follicles^15^,^16^. In particular, autophagy may serve primarily as a pro-death mechanism that aggravates GC injury under stress conditions^17^,^18^. Autophagy selectively removes aggregated proteins (aggrephagy), damaged organelles such as mitochondria (mitophagy) or ribosomes (ribophagy), and invading bacteria (xenophagy) in processes termed selective autophagy^40^,^41^. Mitophagy, the autophagic degradation of mitochondria, has been implicated to facilitate cell death programs^28^. The current study examined whether mitophagy is relevant to the prosurvival effects of FSH on GCs in response to oxidative stress. To our knowledge, this is the first evidence demonstrating a role of mitophagy in FSH-mediated GC protection. Therefore, these findings may bring forward brand new aspects involving mitophagic GC death for future studies in follicular atresia.

Autophagy and apoptosis show distinct interactions depending on cell types or different stimuli^42^. Recent evidence from several mammalian cells implicates the activation of autophagic death without triggering apoptosis under oxidative stress conditions^43^,^44^. Although the induction of apoptotic PCD was observed in GCs with chronic H_2_O_2_ incubation^26^, the current data suggest that the apoptosis-independent mitophagic death functions predominantly in the early stages of oxidative stress-induced GC injury. This suggests that GC suffering short-term oxidative stress may preferentially die via the mitophagic pathway. Conversely, long-term oxidative stress tends to induce GC death via the apoptotic PCD. Nevertheless, our study might shed new light on the switch mechanism of mitophagic/apoptotic GC death upon oxidative stimulation.

PTEN-induced putative kinase 1 (PINK1) is a mitochondrial serine/threonine protein kinase that plays critical roles in regulating mitochondrial dynamics, trafficking, and quality control^45^,^46^. Generally, the mitochondrial responses to PINK1 vary according to the condition-specific trigger^45^. For example, PINK1 may promote the intramitochondrial repair of mitochondrial DNA (mtDNA) under mild mitochondrial stress. In contrast, when mitochondria are severely damaged, PINK1 targets the depolarized mitochondria for mitophagic elimination^45^. However, it is still unknown whether PINK1 is involved with the FSH-mediated protection of GCs from oxidative injury. Here, we found that the suppression of PINK1 induction by FSH treatment or RNAi silencing protected against mitochondrial clearance, which was followed by the restoration of GC viability upon oxidative stress, suggesting that the downregulation of mitophagic death in GCs treated with FSH is associated with PINK1 dysfunction.

FSH promotes GC survival by coordinating multiple downstream effectors^47^. However, the mechanisms that regulate mitophagy in response to FSH signaling remain unclear. Several studies have shown that Parkin, the E3 ubiquitin ligase identified as a key modulator of mitochondrial quality control, facilitates the PINK1-directed autophagic clearance of depolarized mitochondria^24^,^25^. Since decreased mitochondrial membrane potential (Δψm) prevents proteasomal degradation of PINK1, it was proposed that PINK1 becomes stabilized on damaged mitochondria to activate Parkin^23^. Given the role of FSH in suppressing the loss of Δψm and mitophagic GC death under oxidative stress conditions (Figs 3 and 5), we investigated whether the PINK1-Parkin pathway is involved. The results suggest that PINK1 stimulates Parkin-associated mitochondrial clearance in GCs suffering oxidative injury. In contrast, the suppression of PINK1 by FSH leads to the dissociation of Parkin from mitochondria, which is associated with impaired mitophagic activity and increased cellular resistance to oxidative damage. Collectively, this work may provide the first evidence linking the PINK1-Parkin-mitophagy axis to FSH-mediated GC protection.

In summary, we demonstrated that FSH suppresses mitophagy via the PINK1-Parkin cascade to promote GC survival from oxidative injury. Targeting this pathway using small molecule PINK1 inhibitors might provide benefits to clinical therapy for anovulatory disorders such as polycystic ovarian syndrome and premature ovarian failure.

## Materials and Methods

### Animals and ovary collection

All procedures with mice were conducted in accordance with the guidelines of the Animal Research Institute Committee at Nanjing Agricultural University. Three week-old female ICR mice (Qing Long Shan Co.; Animal Breeding Center Nanjing; China) were injected by intraperitoneally with 10 IU pregnant mare serum gonadotropin (PMSG). After 48 hours, all mice were sacrificed by cervical dislocation, and their ovaries were harvested for *in vitro* experiments. Prior to sacrifice, the mice were housed five per cage in a temperature-controlled (22 ± 2 °C) room with a 12: 12 h light: dark cycles (lights on from 7:00 a.m. to 7:00 p.m.); they had *ad libitum* access to water and food. All experimental protocols were approved by the Committee of Animal Research Institute, Nanjing Agricultural University, China.

### Cell culture and treatments

Primary GC cultures were performed as described^26^. Briefly, superovulated mouse ovaries were harvested and individually transferred into 35 mm Petri dishes containing PBS, and GCs were collected by follicle puncture under a surgical dissecting microscope. The cells were cultured in DMEM/F-12 (1:1) medium (Invitrogen) supplemented with 10% fetal bovine serum (Gibco), 100 U/ml penicillin, and 100 μg/ml streptomycin (Gibco) for 4 days at 37 °C with 5% CO_2_. For FSH treatment, GCs exposed to 200 μM H_2_O_2_ (Sigma) for 0 or 1 h were rinsed with PBS and grown in serum-free DMEM/F-12 containing 7.5 IU/ml FSH (ProSpecbio, Ness-Ziona, Israel) for 0.5, 1, 2, or 3 h, as indicated. In some experiments, GCs were treated with 50 μM chloroquine (C6628; Sigma), 10 μM cyclosporine A (9973; Cell Signaling Technology), or 50 μM Z-VAD-FMK (S7023; Selleckchem) for 1, 2, or 3 h following incubation with H_2_O_2_ for 1 h. For RNA interference, GCs were transfected with PINK1 siRNA (sc-44599; Santa Cruz) or scrambled control siRNA (sc-37007; Santa Cruz) for 24 h. After incubation with 200 μM H_2_O_2_ for 0 or 1 h, the GCs were rinsed with PBS and cultured in serum-free medium with or without 7.5 IU/ml FSH for 2 h.

### Detecting acidic vesicular organelles (AVOs) with acridine orange

Autophagy involves the sequestration of cytosol or cytoplasmic organelles into double membranes, which is characterized by the formation of acidic vesicular organelles (AVOs)^48^. Cells exposed to H_2_O_2_ incubation (200 μM) for 1 h were treated with 7.5 IU/ml FSH for 2 h, and then stained with 1 μg/ml acridine orange for 15 min. The emission of green (510–530 nm) and red (650 nm) fluorescence from representative fields was visualized using a Zeiss LSM 710 laser-scanning confocal microscope.

### Measuring autophagosome formation

The GFP-tagged microtubule-associated protein 1 light chain 3 (LC3) expression vector was kindly provided by Prof. Jayanta Debnath (Addgene plasmid, 22418). The GFP-LC3 expression plasmid was transfected into GCs grown on coverslips. After 48 h, cells were exposed to 200 μM H_2_O_2_ for 1 h and then treated with 7.5 IU/ml FSH for another 2 h. The distribution and fluorescence emitted by GFP-LC3 puncta were then observed under a laser-scanning confocal microscope (Zeiss LSM 710 META; Carl Zeiss; Germany). Experiments were repeated three times, and GFP-LC3-stained puncta were counted in three randomly selected fields from each coverslip.

### Cell viability assay

Cell viability was assessed by measuring the conversion of tetrazolium salt (WST-8) to formazan according to the manufacturer’s instructions (Dojindo Laboratories; Kumamoto; Japan). Briefly, cells seeded in 96-well plate were exposed to H_2_O_2_ and grown in the presence of FSH or mitophagy inhibitors for the indicated time periods. After treatment, 10 μl of CCK-8 solution was added to each well containing 100 μL medium, and incubated for an additional 2 h at 37 °C. Cell viability was then determined by reading the optical density at 450 nm using a microplate reader (Bio-Rad; Hercules, CA, USA).

### TMRM/MitoTracker Green double staining

Changes in mitochondrial membrane potential (Δψm) were detected using the fluorescent dye tetramethylrhodamine methyl ester (TMRM) as described previously^49^. Briefly, GCs grown on glass coverslips were treated as indicated and then exposed to 20 nM TMRM and 50 nM MitoTracker Green (Molecular Probes) for 30 min at 37 °C. Confocal images were acquired using a Zeiss LSM 710. The ratio of red (TMRM) to green (Mitotracker) fluorescence intensity served as an indicator of Δψm.

### JC-1 staining to determine Δψm

GCs grown on coverslips were incubated with 0.1 μM JC-1 (Molecular Probes) for 20 min at 37 °C. JC-1 selectively enters mitochondria as aggregates and emits red fluorescence with an excitation at 525 nm. When mitochondrial membrane potential is lost, JC-1 is released to the cytosol and forms a monomer that emits green fluorescence when excited at 490 nm. Cells treated with CCCP (50 mM) for 20 min before JC-1 staining served as a positive control. Fluorescence images were captured using a laser-scanning confocal microscope (Zeiss LSM 710 META); Δψm was quantified by calculating the ratio of aggregated JC-1 (red fluorescence) to monomeric JC-1 (green fluorescence).

### Transmission electron microscopy

Cultured GCs exposed to the treatments described above were harvested and pelleted by centrifugation at 2000 rpm for 15 min. Specimens were prepared for transmission electron microscopy under the supervision of the Imaging Facility of Nanjing Agricultural University. Briefly, the cell mass (~1 mm^3^) was fixed with 2.5% (vol/vol) glutaraldehyde in phosphate buffered saline (PBS; 4 °C, pH 7.4, 0.1 M) for 24 h. After fixation, the samples were post-fixed with 1% osmium tetroxide, dehydrated, and embedded in Araldite. The cell pellets were sectioned to ~50 nm, mounted on Formvar-coated grids, and contrasted with uranyl acetate. Representative areas were selected for imaging using transmission electron microscopy (TEM; Hitachi H-7650; Hitachi) at an accelerating voltage of 80 kV.

### Confocal imaging of mitophagic vacuoles

To analyze the distribution of mitochondria in autophagic vacuoles, GCs were treated as indicated and exposed to sequential double staining with MitoTracker Red and Dansyl monocadaverin (MDC, Sigma). Briefly, the mitochondria were labeled by culturing cells with 50 nM MitoTracker Red at 37 °C for 20 min. Autophagic vacuoles were then incubated with 50 μM MDC for 10 min at 37 °C. After being washed twice with PBS, the cells were immediately observed under a laser-scanning confocal microscope (Zeiss LSM 710 META).

### Visualizing mitophagy by Keima transfection

The pH-sensitive fluorescent protein Keima was used as a specific probe to quantify mitophagy^50^. GCs were seeded on coverslips in 12-well plates, grown to 70% confluence, and transfected with pMT-mKeima-Red (AM-V0251, MBL, Japan) using Lipofectamine 3000 (Invitrogen). Twenty-four hours later, cells were exposed to 200 μM H_2_O_2_ for 1 h, rinsed with PBS, cultured for another 3 h in the presence of 7.5 IU/ml FSH, and then examined under a laser-scanning confocal microscope (Zeiss LSM 710 META; Carl Zeiss; Germany). Mitochondria-localized Keima emits red fluorescence (pseudo-colored) after excitation at 440 nm, whereas acidic Keima puncta emit green fluorescence (pseudo colored) with excitation at 590 nm in autolysosomes. The mitophagic activity was quantified by calculating the number of green puncta (acidic Keima) per cell. Experiments were repeated three times, and acidic Keima dots were counted in three randomly selected fields from each coverslip.

### RNA interference

siRNA specific for PINK1 (sc-44599) and its scrambled controls (sc-37007) were purchased from Santa Cruz Biotechnology. siRNA transfections were performed using Lipofectamine 3000 reagent (Invitrogen) according to the manufacturer’s instructions.

### Measuring caspase-3 activity

Caspase-3 activity was determined using a caspase-3 Activity Assay Kit (Beyotime) as previously described^51^. Briefly, cytosolic extracts were centrifuged at 20,000 × *g* at 4 °C for 15 min, and the protein concentrations were determined using a Bradford Protein Assay Kit (Beyotime). Ten micrograms of total protein were incubated with 100 μl fluorogenic substrate (Ac-DEVD-pNA; Beyotime) for 2 h at 37 °C. The cleavage of Ac-DEVD-pNA was then detected at 405 nm using a microplate reader (Bio-Rad; Hercules; CA; USA) and the amount of p-nitroaniline yielded per minute was calculated.

### Immunofluorescence

GCs were grown on coverslips in 12-well plates for 4 days and then exposed to the treatments described above. After washing in PBS, the cell climbing sheets were fixed with 4% paraformaldehyde (PFA) for 1 h, permeabilized using 0.5% Triton X-100 in PBS for 10 min at 4 °C, and blocked with 1% BSA for 1 h at room temperature. The coverslips were then incubated with 1:100 dilutions of anti-Tom20 (sc-11415; Santa Cruz), anti-Parkin (sc-32282; Santa Cruz), or anti-LC3 (L7543; Sigma) for 1 h at 37 °C. They were subsequently stained for another 1 h with rabbit or mouse Alexa Fluor 488 (A-11008), 568 (A-11031), and 633 (A-21072) secondary antibodies at 1:200 dilutions (Invitrogen). The coverslips were washed, mounted on slides, and observed under a laser-scanning confocal microscope (Zeiss LSM 710 META).

### Western blotting

Total protein extracts were fractioned by electrophoresis on 12% ExpressPlus^TM^ PAGE gels (Genscript) and transferred to PVDF membranes (Millipore, Billerica, MA, USA) by electroblotting. Non-specific binding sites were blocked with 5% bovine serum albumin in TBST for 1 h. The membranes were then incubated with primary antibodies against LC3 (L7543; Sigma), α-tubulin (T5168; Sigma), p62 (ab101266; Abcam), p-mTOR (5536; Cell Signaling Technology), caspase-3 (sc-7148; Santa Cruz), Tom-20 (sc-11415; Santa Cruz), and PINK1 (sc-33796; Santa Cruz) diluted 1:1000 in blocking solution overnight. They were then incubated with horseradish peroxidase-conjugated secondary antibodies (1:2000) for another 1 h. Bands were visualized using SuperSignal West Pico chemiluminescent substrate (Pierce; Rockford; IL; USA).

### Statistical analysis

Statistical significance was determined using SPSS version 16.0 software. All experiments were repeated at least three times. Data are presented as means ± S.E. Pairwise comparisons were assessed using Student’s *t*-tests, and p-values < 0.05 were considered to be statistically significant.

## Additional Information

**How to cite this article**: Shen, M. *et al*. FSH protects mouse granulosa cells from oxidative damage by repressing mitophagy. *Sci. Rep.* **6**, 38090; doi: 10.1038/srep38090 (2016).

**Publisher's note:** Springer Nature remains neutral with regard to jurisdictional claims in published maps and institutional affiliations.

## Supplementary Material

Supplementary Material
