# Supplementary material for: FSH protects mouse granulosa cells from oxidative damage by repressing mitophagy
Source: Sci Rep. 2016 Nov 30;6:38090. doi: 10.1038/srep38090 (PMC5128862; doi:10.1038/srep38090)
Supplement: Supplementary Material [file srep38090-s1.doc]

**Title: FSH protects mouse granulosa cells from oxidative damage by repressing mitophagy**

**Running title: FSH reduces mitophagic granulosa cell death**

Ming Shen^1*^, Yi Jiang^1ab^, Zhiqiang Guan^1a^, Yan Cao^1a^, Shao-chen Sun^1a^ and Honglin Liu^1*^

^1^College of Animal Science and Technology, Nanjing Agricultural University, Nanjing 210095, China

^a^Co-authors:

1. Yi Jiang

Tel: 15996209564

E-mail: 2014105076@njau.edu.cn

2. Zhiqiang Guan

Tel: 15295583971

E-mail: 15110125@njau.edu.cn

3. Yan Cao

Tel: 18801587112

E-mail: 2015105014@njau.edu.cn

4. Shao-chen Sun

Tel: 025-84399092

E-mail: sunsc@njau.edu.cn

*Correspondence author:

1. Ming Shen

Tel: 13585117208

E-mail: shenm2015@njau.edu.com

2. Honglin Liu

Tel/Fax: +86 25 84395106

E-mail: liuhonglin@njau.edu.cn

^b^Co-first author.


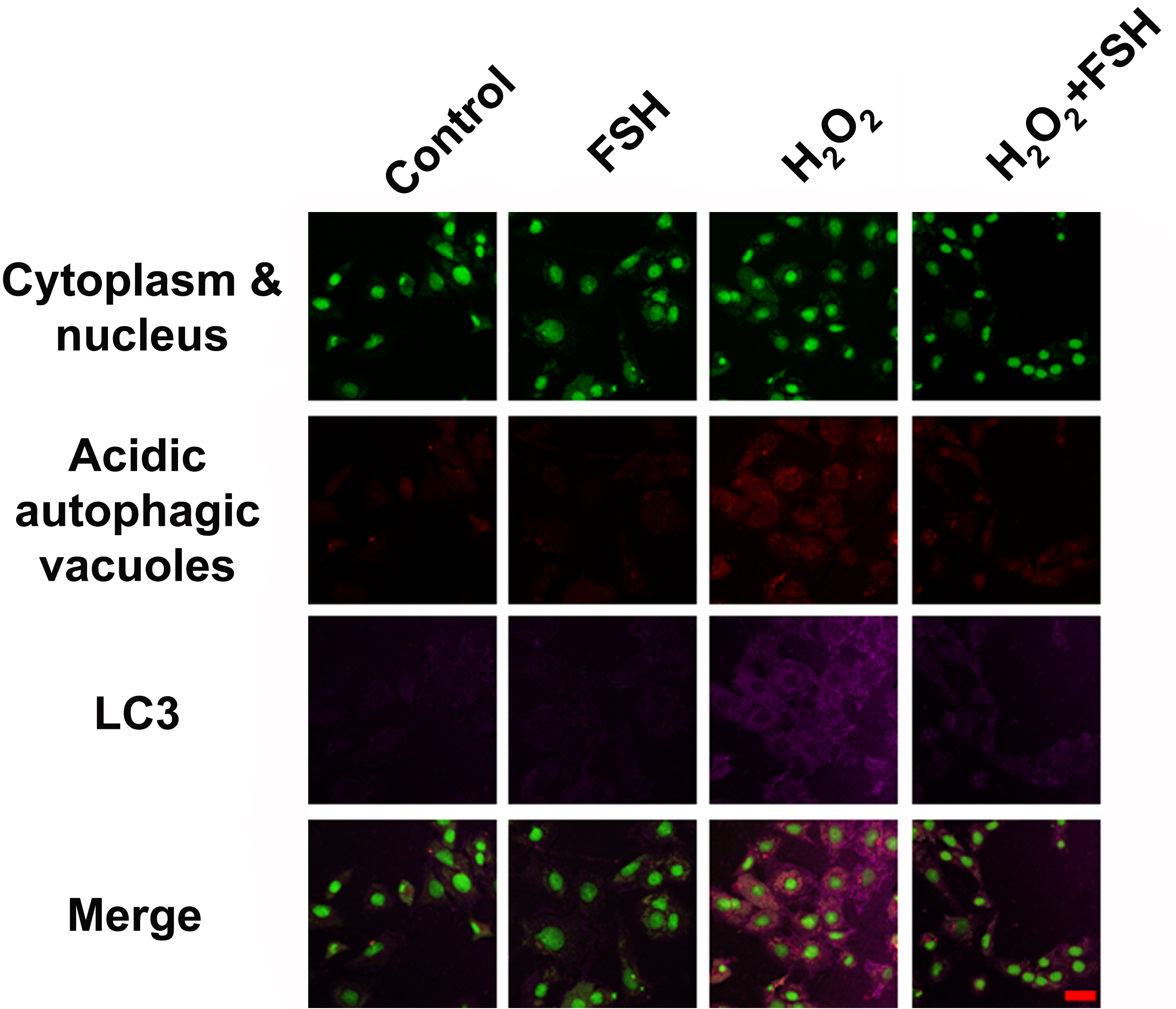


**Supplementary Fig. 1. The induction of AVOs formation and LC3 expression by H_2_O_2_ exposure was inhibited in FSH-treated GCs.** Primary cultured GCs were exposed to 200 μM H_2_O_2_ for 1 h and then treated with 7.5 IU/ml FSH for 2 h. The acidic autophagic vacuoles (AVOs, red) were detected using acridine orange staining, and the LC3 protein was counterstained with anti-LC3 (pseudo coloured; purple). Bar, 20 μm.


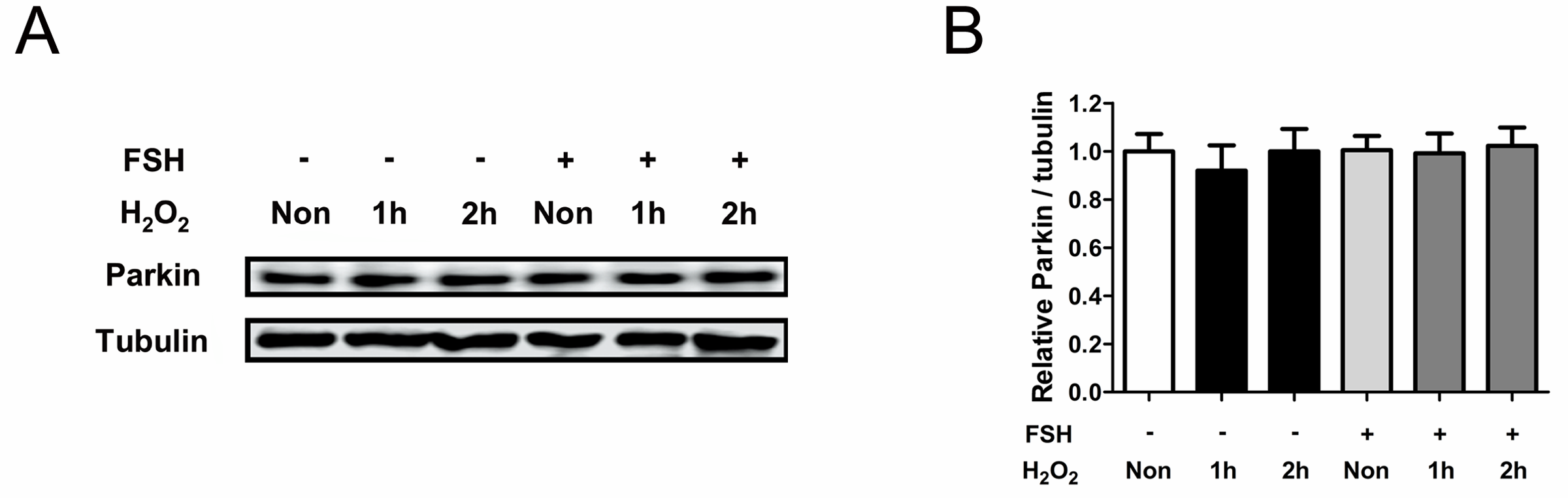


**Supplementary Fig. 2. FSH did not significantly change the expression of Parkin in H_2_O_2_-treated GCs.** (A) GCs incubated with or without 200 μM H_2_O_2_ for 1 h were then cultured in medium containing 7.5 IU/ml FSH for 1-2 h. The expression of Parkin was determined by western blotting. (B) Quantification of relative Parkin protein levels using densitometric analysis. α-tubulin served as the control for loading. Data represent mean ± S.E; n = 3.
